# RETRACTION: Platelet‐Rich Plasma Influence on the Sternal Wounds Healing: A Meta‐Analysis

**DOI:** 10.1111/iwj.70183

**Published:** 2025-01-12

## Abstract

**RETRACTION**: ZhuS. , GaoJ. , YuW. , XiongJ. , “Platelet‐Rich Plasma Influence on the Sternal Wounds Healing: A Meta‐Analysis,” International Wound Journal 20, no. 9 (2023): 3794–3801, 10.1111/iwj.14279.37350616 PMC10588320

The above article, published online on 23 June 2023, in Wiley Online Library (http://onlinelibrary.wiley.com/), has been retracted by agreement between the journal Editor in Chief, Professor Keith Harding; and John Wiley & Sons Ltd. Following an investigation by the publisher, all parties have concluded that this article was accepted solely on the basis of a compromised peer review process. The editors have therefore decided to retract the article. Author S. Zhu disagrees with the retraction. All other authors did not respond to the notice regarding the retraction.

**RETRACTION**: S. Zhu , J. Gao , W. Yu , J. Xiong , “Platelet‐Rich Plasma Influence on the Sternal Wounds Healing: A Meta‐Analysis,” International Wound Journal 20, no. 9 (2023): 3794–3801, 10.1111/iwj.14279.37350616 PMC10588320

The above article, published online on 23 June 2023, in Wiley Online Library (http://onlinelibrary.wiley.com/), has been retracted by agreement between the journal Editor in Chief, Professor Keith Harding; and John Wiley & Sons Ltd. Following an investigation by the publisher, all parties have concluded that this article was accepted solely on the basis of a compromised peer review process. The editors have therefore decided to retract the article. Author S. Zhu disagrees with the retraction. All other authors did not respond to the notice regarding the retraction.

